# Prevalence of restless legs syndrome in Pakistan: a systematic review and meta-analysis across diverse study populations

**DOI:** 10.1186/s12883-026-04981-8

**Published:** 2026-06-15

**Authors:** Hamideh Ebrahimi, Maryam Shaghaghi Ranjbar, Nazanin Ebrahimi, Shima Sadat Aghahosseini

**Affiliations:** 1 Lahore School of Nursing, The University of Lahore, Lahore, Pakistan; 2 Department of Nursing, TeMS.C, Islamic Azad University, Tehran, Iran; 3 Statistics Expert, Student Research Committee, University of Qom, Qom, Iran

**Keywords:** Restless Legs Syndrome, Prevalence, Systematic Review, Meta-analysis, Pakistan

## Abstract

**Background:**

Restless Legs Syndrome (RLS) is a common sleep-related disorder that negatively affects sleep and quality of life. This study aimed to synthesize prevalence across diverse Pakistani populations of RLS in Pakistan and compare it across different populations and regions.

**Methods:**

This systematic review and meta-analysis was conducted in accordance with the PRISMA 2020 guidelines, and the study protocol was registered in the PROSPERO database (registration number: CRD420261319739). The databases PakMediNet, PubMed, Embase, Scopus, Web of Science, and Google Scholar were searched up to February 2026. Observational studies reporting the prevalence of RLS using validated diagnostic tools were included. Study quality was assessed using the Joanna Briggs Institute (JBI) critical appraisal tool. A random-effects model was applied to estimate pooled prevalence, and heterogeneity, subgroup analyses, meta-regression, and publication bias were systematically evaluated.

**Results:**

A total of 61 studies comprising 65 independent reports were included in the meta-analysis. Pooled prevalence across included Pakistani study populations was high, with substantial heterogeneity observed across studies. Higher prevalence estimates were identified in several high-risk groups, including pregnant women, patients undergoing hemodialysis, individuals with diabetes, and students. Although most studies employed the IRLSSG diagnostic criteria, heterogeneity remained considerable. Moreover, the geographic distribution of studies was uneven, with no data available from Baluchistan Province.

**Conclusion:**

RLS appears to be a highly prevalent yet under-recognized condition in Pakistan. Population-based studies employing standardized methodologies, broader geographic coverage, and focused investigation of high-risk groups are warranted to achieve more accurate estimates of disease burden and to better inform future research priorities, healthcare awareness, and epidemiological planning.

**Supplementary Information:**

The online version contains supplementary material available at 10.1186/s12883-026-04981-8.

## Introduction

Restless Legs Syndrome (RLS) is a common neurological sensory-motor disorder characterized by an irresistible urge to move the legs, usually accompanied by unpleasant sensations that worsen during rest and in the evening or at night, and are partially relieved by movement [1, 2]. According to the revised diagnostic criteria of the International Restless Legs Syndrome Study Group (IRLSSG), these clinical features form the basis of diagnosis [3]. RLS can substantially impair sleep quality, daytime functioning, psychological well-being, and overall quality of life. Patients frequently experience insomnia, chronic sleep deprivation, fatigue, and reduced social and occupational performance [4].

Globally, Restless Legs Syndrome (RLS) is recognized as an important but often underdiagnosed public health problem. Epidemiological studies have shown that prevalence varies substantially according to the population studied and the diagnostic criteria applied. In a recent worldwide meta-analysis of the general adult population, the adjusted prevalence of RLS after accounting for publication bias was approximately 3%, although higher estimates have been reported in women, older adults, and selected clinical populations [5]. The disorder is generally classified into primary (idiopathic) and secondary forms. Primary RLS is often associated with familial predisposition, whereas secondary RLS may occur in association with pregnancy, iron deficiency anemia, chronic kidney disease, diabetes mellitus, rheumatoid arthritis, and other chronic medical conditions [6] .

Although the exact pathophysiology of Restless Legs Syndrome (RLS) has not been fully elucidated, current evidence supports a multifactorial neurobiological model centered on brain iron deficiency and dysfunction of central dopaminergic pathways. Iron is an essential cofactor for tyrosine hydroxylase, the rate-limiting enzyme in dopamine synthesis, and is also involved in dopamine receptor regulation and neurotransmission [7]. Neuroimaging and cerebrospinal fluid studies have demonstrated reduced brain iron stores, particularly in the substantia nigra, putamen, and thalamic regions, even in some patients without overt peripheral iron deficiency. Impaired iron availability may therefore alter dopamine production, receptor sensitivity, and circadian dopaminergic signaling, which could explain the characteristic evening and nocturnal worsening of symptoms. In addition, chronic inflammatory and metabolic conditions such as chronic kidney disease, diabetes mellitus, and pregnancy may further disturb iron homeostasis, peripheral nerve function, and central neurotransmission, thereby increasing susceptibility to secondary RLS. This biological framework may help explain the elevated prevalence observed in several high-risk Pakistani populations included in the present review[6, 8].

Despite its clinical significance, RLS remains substantially under recognized and undertreated in many low- and middle-income countries. Several studies have shown that many symptomatic individuals never receive a formal diagnosis or treatment, often due to low public awareness and insufficient familiarity of healthcare providers with the disorder [9–11].

Although no nationwide epidemiological surveillance system for RLS currently exists in Pakistan, available evidence suggests that the disorder may be both underdiagnosed and underreported rather than genuinely rare. In Pakistan, this challenge may be further compounded by restricted access to sleep medicine services, variability in diagnostic practices, and the predominance of hospital-based rather than community-based research. At the same time, local studies have reported substantial prevalence in high-risk groups, including 32.8% among patients with chronic kidney disease and 47.5% among pregnant women, suggesting that the true burden may be considerable in vulnerable populations [12, 13]. Therefore, the current uncertainty likely reflects a combination of increased risk in susceptible groups together with insufficient recognition and inconsistent reporting at the population level. currently available studies in Pakistan have reported highly variable prevalence estimates, ranging from 5.3% to 88.1% [14, 15]. Such wide variation may reflect differences in study design, sampled populations, diagnostic criteria, and healthcare access. Consequently, the true epidemiological burden of RLS in Pakistan remains uncertain, and no comprehensive synthesis of the available evidence has been available.

Therefore, this systematic review and meta-analysis was conducted to estimate the pooled prevalence of RLS in Pakistan and to summarize the evidence available from published studies.

## Methods

This systematic review and meta-analysis were conducted and reported in accordance with the Preferred Reporting Items for Systematic Reviews and Meta-Analyses (PRISMA 2020) guidelines [16]. The study protocol was prospectively registered in the International Prospective Register of Systematic Reviews (PROSPERO) [17] under the registration number CRD420261319739. The literature search was conducted without time restrictions up to February 2026, which was defined as the final search date.

### Search strategy

To identify relevant studies, we conducted a search in PakMediNet, PubMed, Scopus, Web of Science, Embase and the academic search engine Google Scholar using the keywords restless legs syndrome, RLS, Willis-Ekbom Disease, and Pakistan, along with various combinations of these terms. In addition to the electronic search, we manually reviewed the reference lists of the included studies to identify any additional relevant articles. For Google Scholar, due to the large number of retrieved records, the search results were screened up to the first 10 pages per year, based on relevance. In addition, grey literature was explored through PakMediNet and manual searching of reference lists of the included studies to identify any additional relevant articles.

The search strategy used in PubMed was as follows: (“Restless Legs Syndrome“[Mesh] OR “Restless Legs“[tiab] OR RLS[tiab] OR “Restless Leg Syndrome“[tiab]) AND (“Prevalence“[Mesh] OR prevalence[tiab] OR incidence[tiab] OR frequency[tiab]) AND (“Pakistan“[Mesh] OR Pakistan[tiab] OR Pakistan[ad]). The complete search syntaxes for all databases are provided in Supplementary File 1.

### Inclusion and exclusion criteria

All observational studies (case–control, cohort, and cross-sectional) published in English from 2000 up to February 2026 that reported the prevalence or frequency of restless legs syndrome (RLS) in Pakistan were eligible for inclusion. In addition, only studies that used validated diagnostic tools for RLS and provided sufficient data to allow calculation of effect sizes were included in the meta-analysis. Studies that were review articles, qualitative research, letters to the editor, case reports, or interventional studies were excluded. Moreover, studies published in languages other than English or those lacking sufficient data for analysis were not included. In cases where multiple publications reported data from the same study, the version with the most comprehensive and detailed information was selected.

### Screening of articles, data extraction, and diagnostic assessment

All relevant articles were independently reviewed by two authors. During this process, key information, including the authors’ names, year of publication, sample size, study location, diagnostic tool for restless legs syndrome, mean age of participants, and target population, was recorded in a pre-designed data extraction form. In cases of disagreement between the two authors, a third author was consulted, and differences were resolved through discussion and consensus. To ensure accuracy in data extraction, a final review was conducted by the senior author.

The diagnosis of Restless Legs Syndrome (RLS) in the studies included in this systematic review was based on validated diagnostic criteria and assessment tools. In the majority of studies, the diagnostic criteria established by the International Restless Legs Syndrome Study Group (IRLSSG) were applied. According to these criteria, RLS is defined by four essential features: (1) an irresistible urge to move the legs, usually accompanied by or caused by uncomfortable and unpleasant sensations (2), symptoms that begin or worsen during periods of rest or inactivity; (3) partial or complete relief of symptoms with movement; and (4) symptoms that occur exclusively or predominantly in the evening or at night [18].

A limited number of studies employed alternative diagnostic tools, including the National Institutes of Health (NIH) criteria, the Restless Legs Syndrome Diagnostic Index (RLS-DI), the Johns Hopkins criteria, or operational definitions combined with clinical evaluation by a physician. The NIH criteria and the Johns Hopkins criteria [19] are based on core clinical features of Restless Legs Syndrome, including an urge to move the legs, worsening of symptoms during rest, partial relief with movement, and circadian variation with evening or nocturnal predominance. The RLS-DI is a structured diagnostic instrument designed to standardize case identification based on validated symptom-based criteria [20]. In some studies, operational definitions combined with physician-based clinical assessment were used to identify RLS cases. In some studies, the diagnostic instrument or assessment method was not clearly specified. Such studies were included only if the reported diagnostic approach was consistent with established clinical descriptions of Restless Legs Syndrome or involved physician-based clinical assessment. Given the potential differences in sensitivity and specificity across various diagnostic approaches, a subgroup analysis based on the type of diagnostic tool was conducted to examine its impact on the estimated prevalence of Restless Legs Syndrome [21].

### Statistical analysis

Statistical analyses were performed using STATA version 17. Because substantial clinical and methodological variability was anticipated across studies, pooled prevalence estimates were calculated using a random-effects model with restricted maximum likelihood (REML) estimation. To stabilize variances when pooling proportions, prevalence estimates were analyzed after logit transformation, and the same transformed scale was also used in meta-regression analyses. Corresponding 95% confidence intervals were calculated using the Wald method based on the transformed scale and then back-transformed for presentation [22]. To assess heterogeneity among studies, Cochran’s Q statistic was applied. The degree of heterogeneity was further quantified using the I² index, which was categorized as follows: low (less than 25%), moderate (between 25 and 50%), high (between 50 and 75%), and very high (more than 75%) [23]. To explore potential sources of heterogeneity and examine how different factors influenced the overall effect size, subgroup analysis and meta-regression were performed [24]. In the subgroup analysis, the prevalence of RLS was examined based on measurement tools, geographic regions, and target populations. In the meta-regression, the association between RLS prevalence and factors such as publication year, was evaluated. Publication bias was assessed using a funnel plot and Egger’s test. Since publication bias can lead to over- or underestimation of the combined effect size, particularly in studies with smaller sample sizes or less significant results, corrective methods were applied. To adjust for potential bias and recover unpublished findings, the trim-and-fill method a nonparametric approach that identifies asymmetry in the funnel plot was used [25].

### Methodological quality assessment

The methodological quality of the included studies was independently assessed by two reviewers using the Joanna Briggs Institute (JBI) Critical Appraisal Checklist for Studies Reporting Prevalence Data. This checklist consists of nine items evaluating key methodological aspects, including sample selection, measurement validity and reliability, identification and control of confounding factors, and appropriateness of statistical analysis. Each item was scored as “Yes” (1 point), “No,” “Unclear,” or “Not applicable” (0 points). Based on the total score, studies were categorized as having high quality (scores ≥ 7), moderate quality (scores 4–6), or low quality (scores ≤ 3). Any discrepancies between reviewers were resolved through discussion or consultation with a third reviewer. Quality assessment was used to inform interpretation of the findings, and no study was excluded solely on the basis of methodological quality [26].

## Results

### Systematic review

The study selection process was conducted in accordance with the PRISMA 2020 guidelines. In the initial search, a total of 315 records were identified, including 115 records from the PubMed, Scopus, Web of Science, and Embase databases, and 200 records from PakMediNet and the Google Scholar search engine. After removing 80 duplicate records, 235 titles and abstracts were screened for eligibility.

During the title and abstract screening stage, 160 articles were excluded due to irrelevance to the study topic, inappropriate study design (such as interventional studies, narrative reviews, case reports, or letters to the editor), or failure to report the prevalence of Restless Legs Syndrome. Consequently, 75 articles were selected for full-text review.

**Fig. 1:**
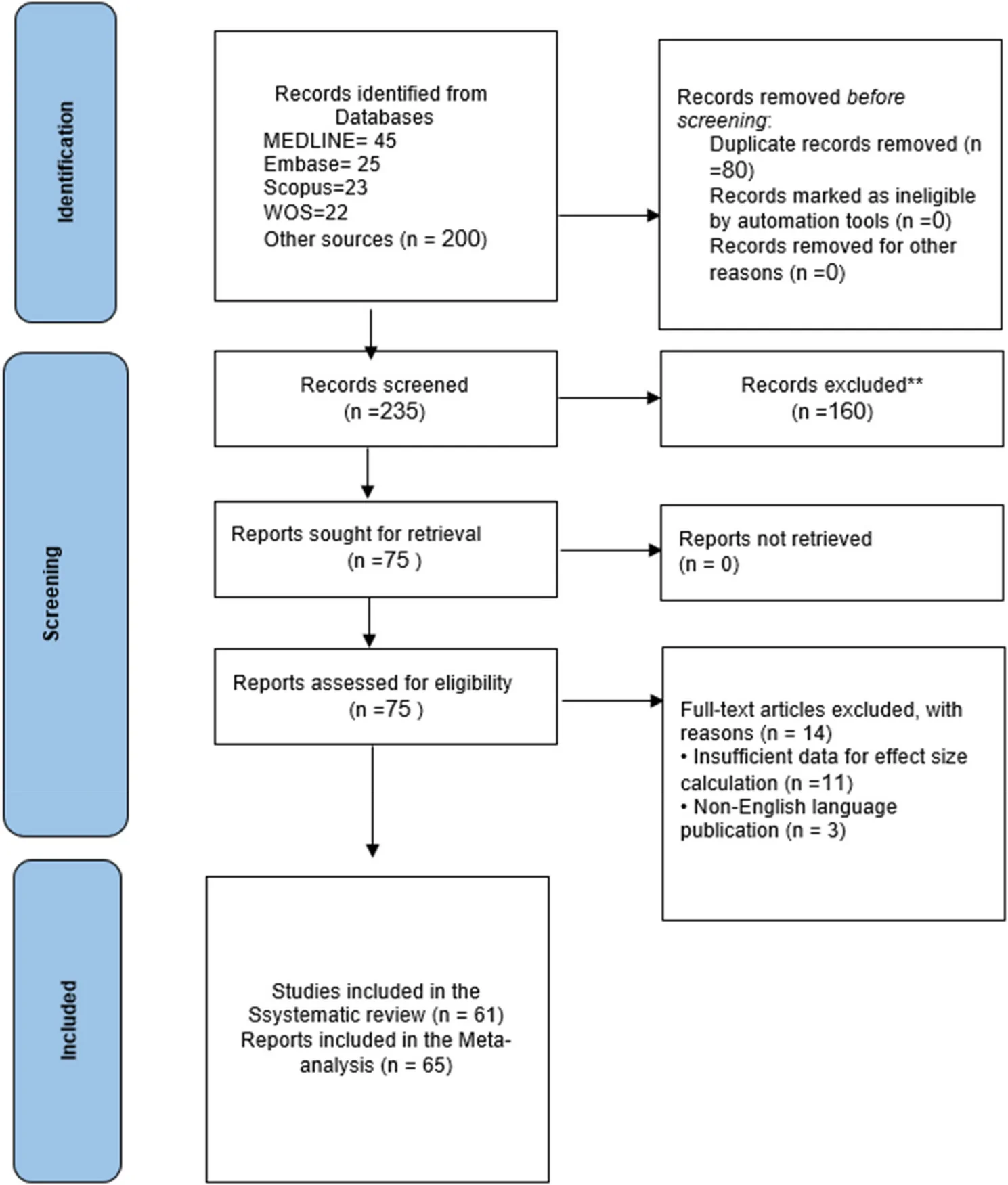
PRISMA flow diagram

At the full-text assessment stage, 14 studies were excluded because of insufficient data for effect size calculation or publication in languages other than English. Ultimately, 61 studies met the eligibility criteria and were included in the systematic review. Among these, several studies reported Restless Legs Syndrome (RLS) prevalence separately for two or more independent target populations or subgroups. Therefore, each eligible subgroup-specific estimate was treated as an independent report for quantitative synthesis, resulting in a total of 65 independent reports derived from 61 studies in the final meta-analysis. The study selection and data extraction process is illustrated in Fig. 1.

Some studies reported the prevalence of Restless Legs Syndrome separately for different subgroups (such as distinct clinical populations or specific underlying conditions). In such cases, each subgroup-specific estimate was treated as an independent analytical unit only when it represented a clearly defined and non-overlapping population. This approach was adopted to enable more precise estimation of prevalence across distinct target groups. However, inclusion of multiple estimates from the same study as independent observations may influence variance estimation and weighting in meta-analysis. Since these subgroup-specific estimates represented distinct populations rather than repeated measurements of the same individuals, this approach was considered methodologically appropriate. Nevertheless, its potential impact on the interpretation of findings was taken into account.

Total of 65 extracted reports from 61 studies were included in the final analysis. The sample size across reports ranged from a minimum of 46 participants to a maximum of 16,371 participants. The mean sample size was 513.9, with a standard deviation of 2,019.0. Regarding diagnostic assessment, the majority of reports used the International Restless Legs Syndrome Study Group (IRLSSG) criteria (56 reports; 86.2%). Other diagnostic tools included the Johns Hopkins criteria, the Restless Legs Syndrome Diagnostic Index (RLS-DI), the National Institutes of Health (NIH) criteria, and operational definitions combined with clinical evaluation, each of which was used in one report (1.5%). In five reports (7.7%), the diagnostic tool was not explicitly specified. In terms of geographic distribution, most reports originated from Punjab Province (33 reports; 50.8%), followed by Sindh Province (23 reports; 35.4%). Additionally, six reports (9.2%) were conducted in Islamabad, and three reports (4.6%) were from Khyber Pakhtunkhwa.

The included studies covered a wide range of target populations. The largest number of reports focused on patients undergoing hemodialysis (15 reports; 23.1%) and pregnant women (15 reports; 18.5%). These were followed by student populations (10 reports; 15.4%) and patients with diabetes mellitus (8 reports; 12.3%). Other groups included patients with hypertension, chronic kidney disease, liver cirrhosis, cardiovascular diseases, spinal cord injury, rheumatoid arthritis, osteoarthritis, individuals with obesity, and various healthy populations, each contributing a smaller proportion of the total reports. The characteristics and findings of the included studies are presented in Table 1.

**Table 1:** Characteristics of studies analyzed for the prevalence of restless legs syndrome in Pakistan

Name	Year	Sample size			Location	Target group	Instrument	Prevalence (%)			Quality
		Total	Male	Female				Total	Male	Female	
Sikandar R [27]	2009	271	-	-	Sindh	Pregnant Women	IRLSSG	30	-	-	Moderate
Ishaq M, Sualeh [28]	2012	240	70	170	Sindh	Patients with RA	IRLSSG	20	5	60	Good
		210	95	115		Healthy People		10	2	19	
Shaikh RA [29]	2014	100	-	-	Sindh	Hemodialysis Patients	Johns Hopkins	32	60	40	Good
Jamal [30]	2014	194	133	61	Punjab	Hemodialysis Patients	IRLSSG	12.4	10.4	16	Good
Haider [31]	2014	250	-	-	Punjab	Hemodialysis Patients	IRLSSG	64.8	56	77.3	Good
Fasih [32]	2015	771	-	-	Sindh	Pregnant Women	IRLSSG	31.9	-	-	Good
Siddiqi [33]	2015	174	93	81	Sindh	Patients with DM	IRLSSG	55.5	-	-	Moderate
Mahmood [34]	2015	390	-	-	Sindh	Outpatients	IRLSSG	23	15.1	30.3	Good
Awan [35]	2017	16,371	-	-	Sindh	Outpatients	N/S	5.3	-	-	Poor
Choudhary [36]	2017	219	87	132	Punjab	Medical Students	IRLSSG	24.6	18	36	Good
Abideen [37]	2018	269	145	134	Islamabad	Hemodialysis Patients	IRLSSG	24	-	-	Good
Shahzad [38]	2018	80	-	-	Punjab	Pregnant Women	IRLSSG	22.5	-	-	Poor
Khan [39]	2019	219	145	74	KPK	Hemodialysis Patients	N/S	32	-	-	Poor
Ishaq M, Riaz [40]	2020	300	80	220	Sindh	Medical students	IRLSSG	8	9.3	8.6	Good
Saleem [41]	2020	137	-	-	Punjab	Pregnant Women	IRLSSG	54	-	-	Good
Memon SF [42]	2020	2117	933	1184	Sindh	Healthy People	IRLSSG	13.4	11.4	14.9	Good
Qureshi [43]	2020	337	40	297	Sindh	Medical Students	IRLSSG	35.6	8.3	91.7	Good
Naqvi [44]	2021	315	-	-	Sindh	Patients with Liver Cirrhosis	IRLSSG	38.4	-	-	Good
Malik [45]	2021	200	100	100	Punjab	Patients with CVD	IRLSSG	14	5	18	Moderate
Yaseen [46]	2021	150	84	66	Sindh	Hemodialysis Patients	IRLSSG	26.6	34.5	16.6	Good
Nawaz [47]	2021	388	188	200	Islamabad	Patients with DM	IRLSSG	81.7	71.2	91.5	Moderate
Mubeen [48]	2022	478	-	-	Sindh	Pregnant Women	IRLSSG	11	-	-	Moderate
Sikandar K [49]	2022	142	48	94	Punjab	Medical Students	IRLSSG	87.3	-	-	Moderate
Ishaq S [50]	2022	162	98	64	Punjab	Hemodialysis Patients	IRLSSG	52.5	45.5	60.5	Moderate
Sagheer [51]	2022	384	-	-	Punjab	Pregnant Women	IRLSSG	63	-	-	Moderate
Moon [52]	2022	150	63	87	Sindh	Hemodialysis Patients	IRLSSG	26.7	12.6	36.7	Good
Masood [53]	2022	75	-	-	Punjab	Patients with DM	IRLSSG	65	-	-	Poor
		75	-	-		Patients with HTN	IRLSSG	49.3	-	-	
		75	-	-		Patients with DM + HTN	IRLSSG	77.3	-	-	
Ibrahim [54]	2022	149	71	78	Punjab	Medical Students	IRLSSG	51	-	-	Good
Shahid. F [55]	2022	335	124	211	Punjab	Patients with DM	IRLSSG	66.2	-	-	Good
Ali [56]	2022	99	66	44	Sindh	Hemodialysis Patients	IRLSSG	27.6			Good
Sultan [57]	2022	112	-	-	Sindh	Hemodialysis Patients	IRLSSG	38.4	53.5	46.5	Good
Siddique [58]	2022	74	54	21	Punjab	Hemodialysis Patients	IRLSSG	38.7	-	-	Moderate
Shaheen [59]	2023	46	-	-	Punjab	Pregnant Women	IRLSSG	43.5	-	-	Moderate
Malik [60]	2023	132	31	101	Punjab	Obese patients	IRLSSG	43.2	-	-	Good
Jahan [61]	2023	200	119	81	Sindh	Medical students	IRLSSG	62.2	-	-	Moderate
Ijaz [15]	2023	151	78	73	Punjab	Hemodialysis Patients	IRLSSG	88.1	-	-	Moderate
Zafar [62]	2023	75	-	-	Punjab	Pregnant Women	IRLSSG	97.3	-	-	Moderate
Pervez [63]	2023	70	62.2	37.8	Punjab	Hemodialysis Patients	IRLSSG	52.8	-	-	Moderate
Afzal [64]	2023	191	71	121	Islamabad	Students	IRLSSG	14.1	10	17	Moderate
Akram [65]	2023	424	214	210	Punjab	Medical Students	N/S	65.8	-	-	Moderate
Gillani [66]	2023	253	128	125	Punjab	Patients with SCI	IRLSSG	45.8	55.2	44.8	Good
Manan [67]	2023	57	27	30	KPK	Healthy People	IRLSSG	33	-	-	Moderate
Manan [67]	2023	57	24	33	KPK	Patients with DM	IRLSSG	8.8			Moderate
Khalid [68]	2023	144	64	80	Punjab	Patients with DM	IRLSSG	71.5	34	29	Good
Khanzada [69]	2024	354	-	-	Punjab	Madrassa Students	IRLSSG	57	37	63	Good
Shaikh NJ [70]	2024	200	-	-	Sindh	IT Student	IRLSSG	78.5	57.5	42.5	Moderate
Rehman [71]	2024	178	-	-	Punjab	Medical Students	IRLSSG	36	-	-	Good
Butt [72]	2024	225	-	-	Punjab	Patients with DM	N/S	13.3	10.4	15.7	Moderate
Qaiser [73]	2024	250	-	-	Punjab	Pregnant Women	IRLSSG	82.9	-	-	Good
Ishaq M, Mahesh [74]	2024	102	-	-	Sindh	Pregnant Women	IRLSSG	36.5	-	-	Good
Yaseen [75]	2024	385	-	-	Islamabad	Patients with RA	N/S	15.6	-	-	Moderate
Basit [76]	2024	280	-	-	Punjab	Hemodialysis Patients	IRLSSG	15.7	-	-	Good
Nawazish [77]	2025	88	52	36	Punjab	Hemodialysis Patients	IRLSSG	46.6	36.5	61.1	Moderate
Memon Z [12]	2025	204	0	204	Sindh	Pregnant Women	IRLSSG	47.5	0	47.5	Moderate
Ghauri [78]	2025	260	117	143	Sindh	Osteoarthritis	IRLSSG	74.2	70.90	76.9	Moderate
Saleem [13]	2025	250	176	74	Punjab	CKD	Operational definition And Clinical evaluation	32.8	33.5	31.3	Moderate
Shahid A [79]	2025	283	65	218	Punjab	Medical students	IRLSSG	95.8	-	-	Moderate
Saadat [80]	2025	200	114	86	Islamabad	CKD	IRLSSG	32			Moderate
Mehmood [81]	2025	400	-	-	Punjab	T2DM	RLS-DI Restless Legs Syndrome Diagnostic Index	18	-	-	Moderate
Singla [82]	2025	300	135	165	Islamabad	Medical students	IRLSSG	11.2	-	-	Moderate
Feroz [83]	2025	1230	0	1230	Sindh	Pregnant Women	IRLSSG	4.7	-	-	Good
Waris [84]	2026	406	102	304	Punjab	Hypertension	National Institutes of Health (NIH) criteria	15.8	-	-	Good

*RA* Rheumatoid arthritis, *DM* Diabetes, *HTN* Hypertension, *SCI* Spinal Cord Injury, *CVD* Cardio Vascular Disease, *CKD* Chronic Kidney Diseases, *N/S* Non-Specified

### Meta-analysis

#### Aggregate prevalence across included study populations

In total, 65 reports extracted from 61 studies were included in the meta-analysis. The pooled prevalence of Restless Legs Syndrome in Pakistan was estimated to be 40.8% (95% confidence interval [CI]: 34.6%–46.9%). Very high heterogeneity was observed among the included reports (I² = 99.57%, τ² = 0.062), indicating substantial variability in prevalence estimates across studies. The heterogeneity test was statistically significant (Q = 18,043.69; *p* < 0.001).

The prevalence reported in individual studies showed a wide range, varying from 4.0% to 97.3%. This broad dispersion is likely attributable to differences in target populations and diagnostic tools used across studies. Despite the high degree of heterogeneity, the pooled prevalence estimate was statistically significant (z = 13.05; *p* < 0.001). However, given the extremely high heterogeneity observed among the included studies, the pooled prevalence should be interpreted with caution. The substantial heterogeneity suggests that the studies differed markedly in terms of population characteristics, clinical conditions of participants, sampling strategies, and diagnostic assessment methods. Therefore, the overall prevalence estimate should not be interpreted as a precise or uniform national prevalence for the entire population of Pakistan, but rather as an average prevalence across diverse study settings and populations.

In this context, subgroup analyses play a critical role in clarifying potential sources of heterogeneity and enabling a more nuanced interpretation of the prevalence patterns of Restless Legs Syndrome across different populations. Given the very high heterogeneity (I² ≈ 99%), conducting a leave-one-out sensitivity analysis was of limited interpretative value, as the observed heterogeneity was primarily driven by structural differences among studies rather than the influence of any single study. Consequently, the exclusion of individual studies was unlikely to result in meaningful changes in the pooled prevalence estimate or to identify the primary source of heterogeneity. For this reason, the analytical focus was placed on subgroup analyses and meta-regression as more appropriate approaches for exploring potential sources of heterogeneity.

**Fig. 2:**
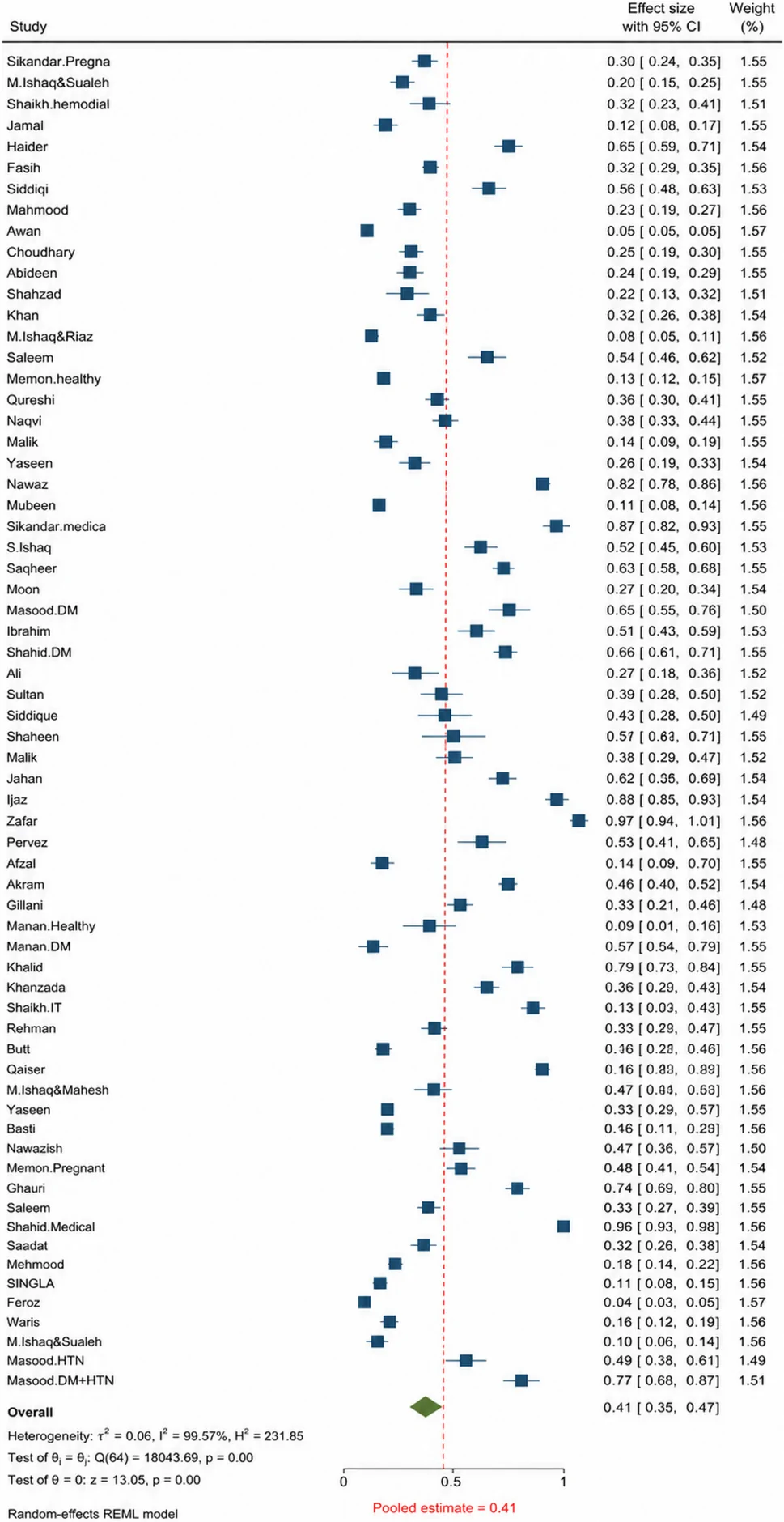
Forest plot showing the pooled prevalence of restless legs syndrome in Pakistan

The forest plot illustrating the pooled prevalence and individual study estimates is presented in Fig. 2.

### Subgroup analyses

It should be noted that the number of studies included in subgroup analyses may differ from the descriptive counts reported above. This is because some studies contributed multiple subgroup-specific estimates or were categorized differently based on the target population. In subgroup analyses, only eligible and non-overlapping study populations relevant to each category were included.

### Prevalence in pregnant women

In the subgroup analysis of pregnant women, 12 studies reporting the prevalence of Restless Legs Syndrome (RLS) in this population were included in the meta-analysis. The pooled prevalence of RLS among pregnant women in Pakistan was estimated at 42.2% (95% CI: 22.6%–64.7%). Heterogeneity among the included studies was very high (I² = 99.14%, τ² = 2.57), and the heterogeneity test was statistically significant (Q = 782.43, *p* < 0.001).

### Hemodialysis patients

In the subgroup analysis of patients undergoing hemodialysis, 15 studies reporting the prevalence of RLS in this population were included in the meta-analysis. The pooled prevalence of RLS among hemodialysis patients in Pakistan was estimated at 37.6% (95% CI: 27.0%–49.5%). Very high heterogeneity was observed among the included studies (I² = 96.34%, τ² = 0.89), and the heterogeneity test was statistically significant (Q = 334.95, *p* < 0.001).

### Students

In the subgroup analysis of student populations, 13 studies reporting the prevalence of RLS among students in Pakistan were included in the meta-analysis. The pooled prevalence of RLS among students was estimated at 48.4% (95% CI: 28.2%–69.1%). Heterogeneity among the included studies was very high (I² = 99.07%, τ² = 2.53), and the heterogeneity test was statistically significant (Q = 708.17, *p* < 0.001).

### Patients with diabetes mellitus

**Fig. 3:**
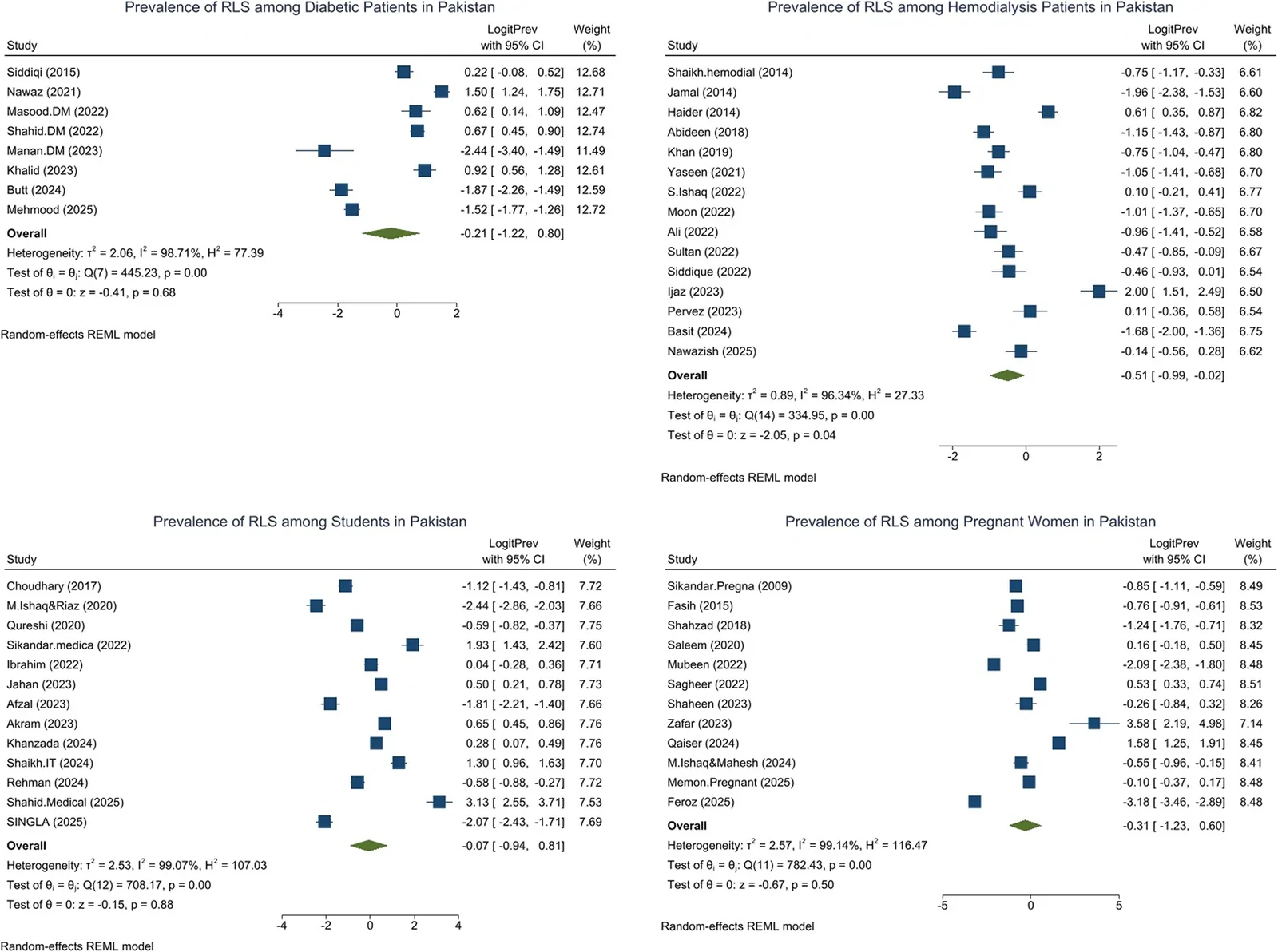
Forest plots of restless legs syndrome prevalence stratified across four predefined subgroup

In the subgroup analysis of patients with diabetes mellitus, 8 studies reporting the prevalence of RLS in this population were included in the meta-analysis. The pooled prevalence of RLS among patients with diabetes in Pakistan was estimated at 44.7% (95% CI: 22.8%–68.9%). Very high heterogeneity was observed among the included studies (I² = 98.71%, τ² = 2.06), and the heterogeneity test was statistically significant (Q = 445.23, *p* < 0.001). Figure 3 illustrates the forest plots of the prevalence of Restless Legs Syndrome across the four predefined subgroups.

### Meta-regression

**Fig. 4:**
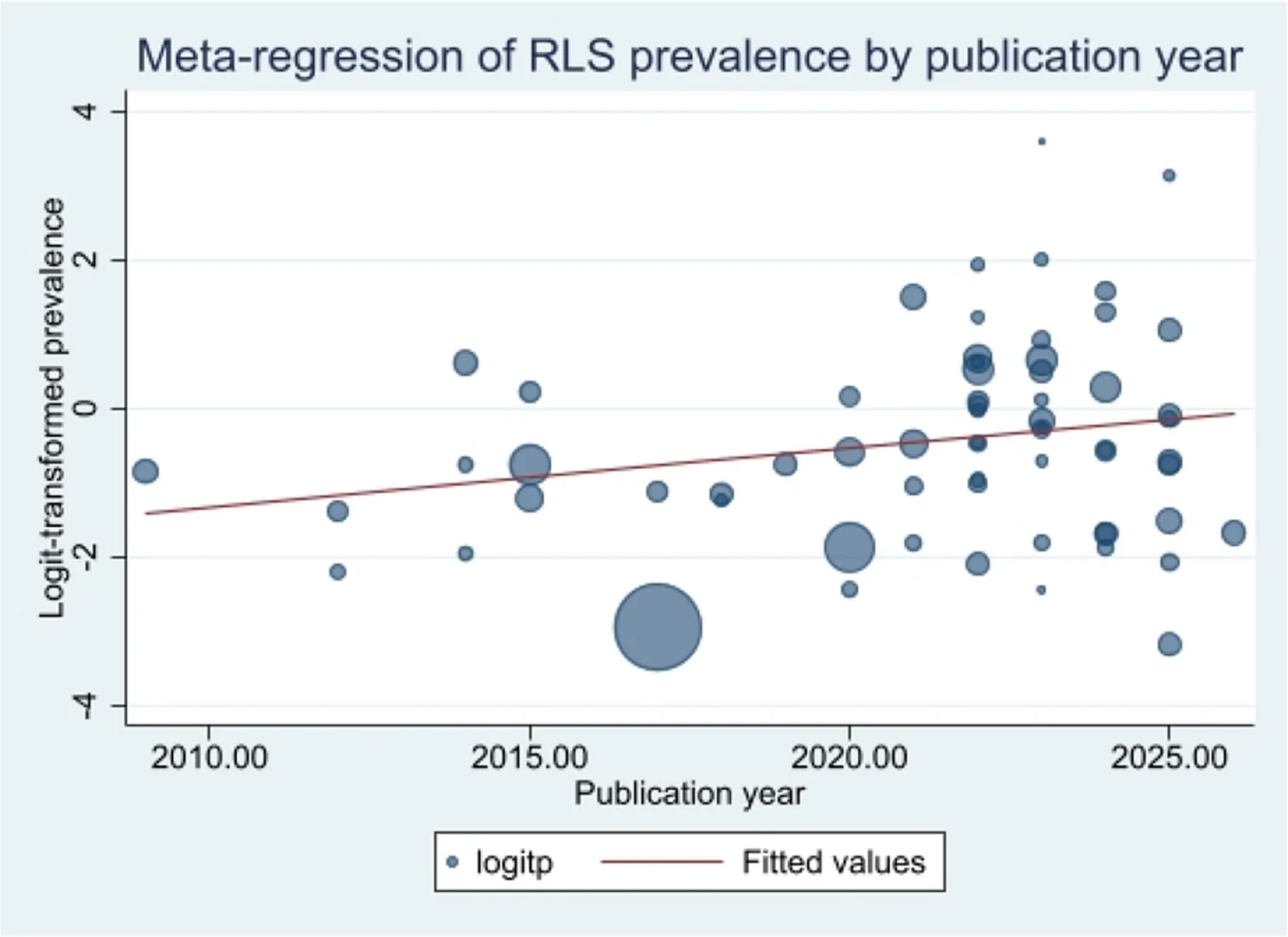
Bubble plot of meta-regression examining the association between publication year and the reported prevalence of restless legs syndrome in Pakistan

To examine the effect of publication year on the reported prevalence of Restless Legs Syndrome, a meta-regression analysis was performed. The results indicated that although the reported prevalence of Restless Legs Syndrome showed a slight increasing trend over time (β = 0.076), this association was not statistically significant (*p* = 0.073). Publication year explained only 3.34% of the between-study heterogeneity, while the residual heterogeneity remained very high (I² = 98.8%). The bubble plot illustrating the meta-regression results is presented in Fig. 4.

### Subgroup analysis based on diagnostic assessment tools

In the subgroup analysis based on diagnostic assessment tools, studies were divided into two groups: those that used the International Restless Legs Syndrome Study Group (IRLSSG) criteria (56 studies) and those that used other diagnostic tools (9 studies). The pooled prevalence of Restless Legs Syndrome in studies based on the IRLSSG criteria was estimated at 41.8% (95% CI: 33.6%–50.4%). In contrast, the pooled prevalence of RLS in studies employing other diagnostic tools was 22.2% (95% CI: 12.8%–35.7%).

In both subgroups, heterogeneity among studies was very high and statistically significant (IRLSSG: I² = 98.62%; other diagnostic tools: I² = 98.81%). The results of this subgroup analysis are presented in Table 2.

**Table 2:** Subgroup analysis of the prevalence of restless legs syndrome according to diagnostic assessment tools and sources of heterogeneity

Diagnostic tool	No. of studies	Pooled prevalence (%)	95% CI (%)	I² (%)	Q (within)	*P* (within)	Q (between)	*P* (between)
IRLSSG criteria	56	41.8	33.6–50.2	98.62	3042.39	< 0.001	7.89	0.005
Other diagnostic tools†	9	22.2	13.0–32.8	98.81	1532.91	< 0.001		

### Subgroup analysis by province

In the subgroup analysis based on province, the included studies were categorized into three groups: Punjab Province (33 studies), Sindh Province (23 studies), and other regions of the country, including Islamabad and Khyber Pakhtunkhwa (9 studies). The pooled prevalence of Restless Legs Syndrome in Punjab Province was estimated at 51.3% (95% CI: 40.3%–62.2%). The pooled prevalence of RLS in Sindh Province was 27.9% (95% CI: 19.4%–38.3%), while in other regions of the country it was 25.4% (95% CI: 13.8%–42.1%).

Across all geographic subgroups, heterogeneity was very high and statistically significant (I² > 97%). The results of this subgroup analysis are presented in Table 3.

**Table 3:** Subgroup analysis by geographic region

Region	No. of Studies	Pooled Prevalence (%)	95% CI (%)	I² (%)	Q (within)	*P* (within)	Q (between)	*P* (between)
Punjab	33	51.3	40.3–62.3	98.37	1287.67	< 0.001	10.61	0.005
Sindh	23	27.9	19.4–37.6	98.99	3078.52	< 0.001		
Other regions*	9	25.4	13.8–38.6	97.79	434.98	< 0.001		

Q (between) and P (between) represent the overall test for differences between subgroups

* Islamabad, Khyber Pakhtunkhwa

### Publication and selection bias

**Fig. 5:**
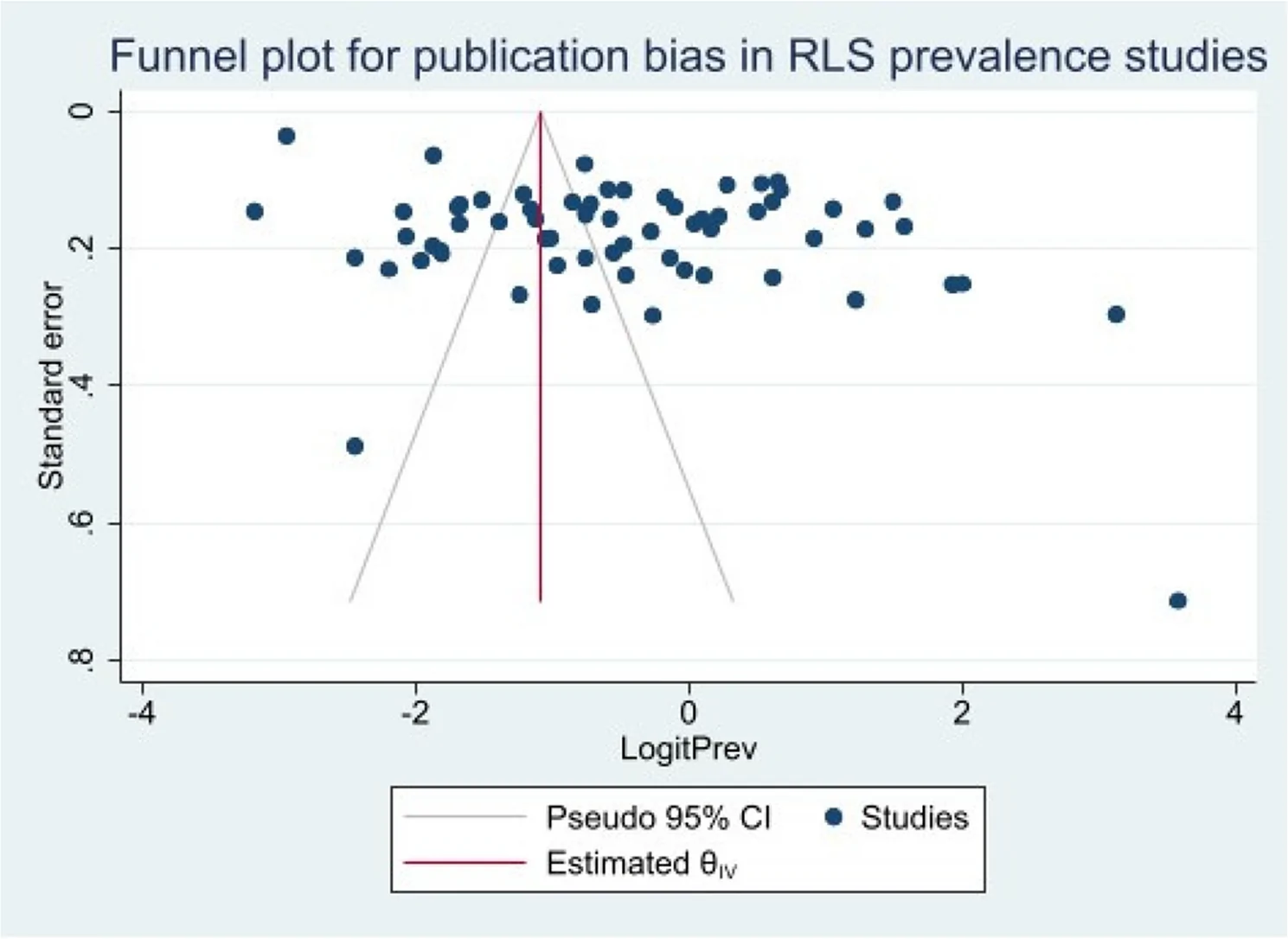
Funnel plot assessing publication bias in studies reporting the prevalence of restless legs syndrome in Pakistan

Publication bias was assessed using Egger’s and Begg’s tests in combination with visual inspection of the funnel plot. Egger’s test indicated a statistically significant small-study effect (z = 2.39, *p* = 0.017), whereas Begg’s test did not provide evidence of publication bias (z = − 0.47, *p* = 0.646). In light of the Egger’s test results, the trim-and-fill method was applied as a sensitivity analysis. This analysis did not impute any hypothetical missing studies, and the pooled prevalence estimate remained unchanged before and after adjustment. However, these findings should be interpreted with caution. In prevalence meta-analyses characterized by extreme heterogeneity and substantial differences in study populations, funnel-plot asymmetry is difficult to interpret, and a significant Egger’s test does not necessarily indicate conventional publication bias alone. Therefore, the observed asymmetry may partly reflect between-study heterogeneity rather than true publication bias. The funnel plot used to assess publication bias is shown in Fig. 5.

With regard to selection bias, the methodological quality assessment using the Joanna Briggs Institute (JBI) checklist revealed that a substantial proportion of the included studies were hospital-based and employed non-random or convenience sampling strategies. In several studies, inclusion criteria and sampling frames were not clearly defined, and study populations were often restricted to specific clinical or high-risk groups. These methodological characteristics increase the likelihood of selection bias and may have led to an overestimation of the reported prevalence of Restless Legs Syndrome compared with the general population. The Critical Appraisal of the articles is presented in Supplementary File 2.

## Discussion

This study represents the first nationwide meta-analysis in Pakistan that synthesizes evidence published between 2000 and February 2026 to estimate the prevalence of Restless Legs Syndrome (RLS) across different regions and demographic/clinical groups. The main finding indicates a pooled prevalence of RLS in Pakistan of 40.8% (95% CI: 34.6–46.9), reflecting a substantial burden of this disorder in the country. However, the presence of very high heterogeneity (I² ≈ 99.6%) suggests that this estimate should be interpreted with caution, as the included studies differed markedly in terms of target populations, diagnostic tools, sampling methods, and participants’ background characteristics. Consequently, the pooled estimate is less representative of a uniform national prevalence in the general population and rather reflects an average prevalence across a heterogeneous set of populations, particularly high-risk and clinical groups.

Comparison with global evidence highlights that prevalence estimates of RLS are highly dependent on the population under study and methodological characteristics. In a worldwide meta-analysis by Broström et al., which focused exclusively on the general adult population, the adjusted prevalence of RLS after accounting for publication bias was approximately 3%. The substantial discrepancy between this estimate and the higher prevalence observed in the present study may be partly explained by the predominantly non–community-based nature of many included Pakistani studies, their focus on high-risk and clinical populations, and differences in diagnostic tools and criteria [5].

The pooled prevalence of RLS among pregnant women in Pakistan was approximately 42–49%, which is considerably higher than global estimates reported in the meta-analysis by Chen et al. That study, including 27 investigations with more than 51,000 pregnant women, reported an overall prevalence of 21%, with a prevalence of 30% in the Eastern Mediterranean region [85]. The marked difference between these figures and the findings of the present study may be explained by differences in population characteristics, nutritional status, prevalence of anemia and iron deficiency, socioeconomic conditions, and the largely hospital-based nature of studies conducted in Pakistan [27]. Despite the numerical differences, the overall pattern is consistent with global evidence indicating that pregnancy is an important condition associated with increased susceptibility to RLS. Iron deficiency should also be emphasized as one of the central biological mechanisms underlying Restless Legs Syndrome. Current evidence suggests that the most consistent pathophysiological abnormality in RLS is reduced brain iron availability, particularly in regions such as the substantia nigra and thalamus, which may occur even in the presence of normal peripheral iron indices. Impaired brain iron homeostasis may alter dopaminergic and glutamatergic neurotransmission, thereby contributing to the sensory and motor symptoms of RLS. This framework is particularly relevant in Pakistan, where iron deficiency anemia remains common in women of reproductive age, pregnant women, and patients with chronic diseases. Clinical studies have further shown that iron supplementation may improve RLS symptoms, sleep quality, and fatigue, supporting the biological relevance of iron status in affected individuals. Therefore, differences in iron deficiency burden across study populations may partly contribute to the substantial variability in prevalence estimates observed in the present meta-analysis [86, 87]. The prevalence of RLS among patients undergoing haemodialysis was also substantially higher than in the general population. A recent systematic review and meta-analysis reported a pooled prevalence of 24.0% (95% CI: 21–26) in this group. That study further demonstrated higher prevalence and severity of RLS among women and in the Americas, suggesting that regional differences, study design, and temporal trends contribute to heterogeneity. From a pathophysiological perspective, mechanisms such as impaired central nervous system iron stores, reduced ferritin levels, and disrupted iron transport to the brain have been proposed in patients with chronic kidney disease and those on hemodialysis. Iron-deficiency anemia related to hemodialysis has also been frequently associated with increased RLS prevalence [88]. Additionally, demographic and ethnic factors appear to play a role; for example, one study reported fewer RLS complaints among African American hemodialysis patients compared with Caucasians, suggesting potential genetic, social, or healthcare access influences [89]. In a study from Sindh province, the prevalence of RLS was approximately 27.6%, and RLS was significantly associated with vitamin D deficiency and longer duration of hemodialysis [56]. Collectively, these findings underscore that RLS is a common and clinically relevant condition in hemodialysis patients, and targeted screening, particularly among women and patients with anemia, iron deficiency, or vitamin D deficiency, may help improve recognition, sleep quality, and overall health outcomes [55].

The findings of the present study also indicate that the prevalence of RLS among patients with type 2 diabetes mellitus is considerably higher than in the general population. Several center-based Pakistani studies have reported RLS prevalence rates of approximately 20–30% in diabetic patients [33, 47, 72]. Diabetes-related factors, including longer disease duration, poor glycemic control, and especially diabetic neuropathy, have been identified as important correlates of RLS. These observations suggest that RLS in diabetic patients may reflect underlying peripheral nervous system involvement. Moreover, Pakistani studies have shown that RLS in diabetic patients is associated with poorer sleep quality[81], increased daytime fatigue, and mental health disturbances [15], which may adversely affect overall diabetes management and quality of life.

An important consideration in interpreting the meta-regression results is the uneven geographic distribution of studies conducted in Pakistan. Most available evidence originates from relatively urbanized and socioeconomically advantaged provinces such as Punjab and Sindh, whereas no studies from Baluchistan were included in the present meta-analysis. This lack of data may influence both the pooled prevalence estimate and the interpretation of regional differences, as Baluchistan differs substantially from other provinces in terms of socioeconomic indicators, access to healthcare services, and nutritional status [90–92]. Therefore, country-level meta-regression results should be interpreted cautiously and may not fully reflect the true pattern of RLS across all regions of Pakistan.

Although the majority of included studies used the standardized diagnostic criteria of the International Restless Legs Syndrome Study Group (IRLSSG), which represents an important methodological strength, substantial heterogeneity persisted. This finding is consistent with previous meta-analyses that reported high heterogeneity despite widespread use of IRLSSG criteria [88, 93]. These observations suggest that the use of a standardized diagnostic tool alone may not fully account for between-study variability. Differences in tool implementation, linguistic and cultural adaptations, inadequate distinction between primary and secondary RLS, and variability in demographic and clinical characteristics of study populations likely play a more prominent role in generating heterogeneity.

Although publication bias was statistically assessed using funnel plots and Egger’s test, the possibility of publication bias cannot be entirely excluded. Many included studies were cross-sectional with relatively small sample sizes and tended to report higher prevalence estimates of RLS, suggesting a potential preference for publishing studies with higher reported prevalence. Conversely, studies reporting lower prevalence or non-significant results may be underrepresented in the published literature.

However, this interpretation should be approached with caution. In the presence of extreme heterogeneity and substantial differences in study populations, funnel-plot asymmetry is difficult to interpret, and a significant Egger’s test does not necessarily indicate conventional publication bias alone.

Quality appraisal using the JBI tool indicated that, although most studies employed valid diagnostic criteria, there were notable weaknesses in identifying and controlling for confounding factors. In many studies, samples were selected non-randomly and predominantly from hospital-based or specific populations, which may introduce selection bias. This issue is particularly relevant in hospital-based studies and investigations involving patients with underlying conditions such as diabetes, chronic kidney disease, or pregnancy, potentially leading to overestimation of the true prevalence of RLS.

Although sleep disturbance and depression were not primary pooled outcomes in the present meta-analysis, these comorbidities remain clinically relevant when interpreting the broader burden of Restless Legs Syndrome (RLS). Previous studies suggest that the relationships between RLS, impaired sleep, and depressive symptoms may be bidirectional rather than strictly unidirectional. Xu et al. reported that 65% of patients with RLS experienced sleep disorders, and poorer sleep quality was significantly correlated with both RLS severity and depression scores [94]. Similarly, Sevim et al., in a population-based study, found significantly higher anxiety and depression symptoms among individuals with RLS compared with controls, with symptom severity correlating with psychiatric symptom scores [95]. In addition, local evidence among patients with type 2 diabetes mellitus demonstrated a significant association between RLS and impaired sleep-related quality of life [96]. Collectively, these findings suggest that RLS may contribute to sleep and psychological disturbances, while poor sleep and depressive symptoms may also amplify symptom perception and overall disease burden. Therefore, these associations should be interpreted cautiously, and causal direction cannot be established from the currently available predominantly cross-sectional evidence.

## Limitations

This study has several important limitations that should be considered when interpreting the findings. First, despite comprehensive search efforts, publication bias cannot be completely excluded, as many included studies were cross-sectional, had relatively small sample sizes, and more frequently reported higher prevalence estimates. Therefore, studies with lower prevalence or non-significant findings may have been less likely to be published or identified. Second, quality assessment using the JBI tool revealed recurrent limitations in the identification and control of confounding variables. Many studies relied on non-random, hospital-based sampling and did not adequately adjust for factors such as age, sex, socioeconomic status, anemia, iron stores, severity of underlying diseases, or other relevant covariates, which may have introduced selection bias and inflated prevalence estimates. Third, the geographic distribution of evidence was uneven, with most studies conducted in Punjab and Sindh and no eligible data from Baluchistan, limiting national representativeness given the marked socioeconomic, nutritional, and healthcare disparities across provinces. Fourth, most included studies did not clearly distinguish between primary (idiopathic) and secondary forms of Restless Legs Syndrome, nor provide sufficient data for separate analyses. Accordingly, the pooled estimates likely reflect a mixture of heterogeneous clinical entities, and findings related to etiological pathways and associated factors should be interpreted cautiously. Fifth, although most studies applied standardized diagnostic criteria (predominantly IRLSSG), some variation in diagnostic instruments and implementation methods remained, which may have contributed to misclassification and residual heterogeneity. Sixth, cultural perceptions of symptoms, language differences, and healthcare-seeking behaviors were rarely reported in the included studies. As RLS is a symptom-based diagnosis, these unmeasured contextual factors may have influenced symptom recognition and prevalence estimates. Seventh, sex-specific biological and social determinants, including hormonal status and pregnancy-related factors, were inconsistently reported, limiting more detailed interpretation of observed gender differences. Finally, the included studies represented diverse populations, ranging from community-based samples and students to high-risk clinical groups such as pregnant women, patients undergoing haemodialysis, and individuals with diabetes mellitus. Consequently, the pooled prevalence is best interpreted as a summary estimate across heterogeneous study populations rather than a precise national prevalence for all Pakistani populations.

## Conclusion

The findings of this meta-analysis suggest that Restless Legs Syndrome is an under-recognized disorder in Pakistan, with a substantial burden observed particularly among the populations most frequently studied, including pregnant women, patients undergoing hemodialysis, individuals with diabetes mellitus, and student groups. These findings indicate that the prevalence of RLS may be notably higher in specific clinical and demographic populations than in the general community. However, because the available evidence was derived from diverse and heterogeneous study populations, the pooled prevalence estimate should be interpreted as an aggregate measure across included populations rather than a precise national prevalence for the general Pakistani population. Available evidence also suggests that RLS may be associated with impaired sleep quality, reduced daily functioning, and lower quality of life in affected individuals. Despite the widespread use of standardized diagnostic criteria in existing studies, methodological variability and uneven geographic coverage warrant cautious interpretation of current estimates. Further well-designed, population-based studies in underrepresented regions are needed to provide a more accurate understanding of the burden of this disorder in Pakistan and may help inform future healthcare planning, physician awareness, and targeted research priorities.

## Supplementary Information

Supplementary Material 1.

Supplementary Material 2.

Supplementary Material 3.

## Data Availability

The data used in this study were obtained from previously published articles and are available in the public domain. No new primary data were generated. All extracted and analyzed data are included in the manuscript and supplementary materials.
