# Supplementary material for: Prevalence of restless legs syndrome in Pakistan: a systematic review and meta-analysis across diverse study populations
Source: BMC Neurol. 2026 Jun 15;26:537. doi: 10.1186/s12883-026-04981-8 (PMC13501774; doi:10.1186/s12883-026-04981-8)
Supplement: Supplementary file 2 [file 12883_2026_4981_MOESM2_ESM.docx]

| **Study** | **Appraised by**  Critical Appraisal Checklist | **Q_1_** | **Q_2_** | **Q_3_** | **Q_4_** | **Q_5_** | **Q_6_** | **Q_7_** | **Q_8_** | **Final Result** |
| --- | --- | --- | --- | --- | --- | --- | --- | --- | --- | --- |
| Awan (11) | **SH.S.A** | Y | U | N | N | U | N | Y | Y | Moderate |
|  |  |  |  |  |  |  |  |  |  |  |
| Ijaz (12) | **SH.S.A** | Y | Y | N | N | U | U | Y | Y | Moderate |
|  |  |  |  |  |  |  |  |  |  |  |
| Sikandar (17) | **SH.S.A** | Y | Y | Y | Y | N | N | Y | Y | High |
|  |  |  |  |  |  |  |  |  |  |  |
| Ishaq (18) | **SH.S.A** | Y | Y | Y | Y | Y | Y | Y | Y | High |
|  |  |  |  |  |  |  |  |  |  |  |
| Shaikh (19) | **SH.S.A** | Y | Y | Y | Y | Y | U | Y | Y | High |
|  |  |  |  |  |  |  |  |  |  |  |
| Jamal (20) | **SH.S.A** | Y | Y | Y | Y | Y | Y | Y | Y | High |
|  |  |  |  |  |  |  |  |  |  |  |
| Haider (21) | **SH.S.A** | Y | Y | Y | Y | Y | Y | Y | Y | High |
|  |  |  |  |  |  |  |  |  |  |  |
| Fasih (22) | **SH.S.A** | Y | Y | Y | Y | Y | Y | Y | Y | High |
|  |  |  |  |  |  |  |  |  |  |  |
| Siddiqi (23) | **SH.S.A** | Y | Y | Y | Y | U | U | Y | Y | Moderate |
|  |  |  |  |  |  |  |  |  |  |  |
| Mahmood (24) | **SH.S.A** | Y | Y | Y | Y | Y | Y | Y | Y | High |
|  |  |  |  |  |  |  |  |  |  |  |
| Choudhary (25) | **SH.S.A** | Y | Y | Y | Y | Y | Y | Y | Y | High |
|  |  |  |  |  |  |  |  |  |  |  |
| Abideen (26) | **SH.S.A** | Y | Y | Y | Y | Y | Y | Y | Y | High |
|  |  |  |  |  |  |  |  |  |  |  |
| Shahzad (27) | **SH.S.A** | Y | U | Y | Y | N | N | Y | Y | Moderate |
|  |  |  |  |  |  |  |  |  |  |  |
| Khan (28) | **SH.S.A** | Y | Y | N | N | U | N | Y | Y | Moderate |
|  |  |  |  |  |  |  |  |  |  |  |
| Ishaq (29) | **SH.S.A** | Y | Y | Y | Y | Y | Y | Y | Y | High |
|  |  |  |  |  |  |  |  |  |  |  |
| Saleem (30) | **SH.S.A** | Y | Y | Y | Y | Y | Y | Y | Y | High |
|  |  |  |  |  |  |  |  |  |  |  |
| Memon (31) | **SH.S.A** | Y | Y | Y | Y | Y | Y | Y | Y | High |
|  |  |  |  |  |  |  |  |  |  |  |
| Qureshi (32) | **SH.S.A** | Y | Y | Y | Y | Y | Y | Y | Y | High |
|  |  |  |  |  |  |  |  |  |  |  |
| Naqvi (33) | **SH.S.A** | Y | Y | N | N | U | U | Y | Y | Moderate |
|  |  |  |  |  |  |  |  |  |  |  |
| Malik (34) | **SH.S.A** | Y | Y | N | N | U | U | Y | Y | Moderate |
|  |  |  |  |  |  |  |  |  |  |  |
| Yaseen (35) | **SH.S.A** | Y | Y | Y | Y | Y | Y | Y | Y | High |
|  |  |  |  |  |  |  |  |  |  |  |
| Nawaz (36) | **SH.S.A** | Y | Y | Y | Y | U | U | Y | Y | Moderate |
|  |  |  |  |  |  |  |  |  |  |  |
| Mubeen (37) | **SH.S.A** | Y | Y | Y | Y | U | N | Y | Y | Moderate |
|  |  |  |  |  |  |  |  |  |  |  |
| Sikandar (38) | **SH.S.A** | Y | Y | Y | Y | U | U | Y | Y | Moderate |
|  |  |  |  |  |  |  |  |  |  |  |
| Ishaq (39) | **SH.S.A** | Y | Y | Y | Y | U | U | Y | Y | Moderate |
|  |  |  |  |  |  |  |  |  |  |  |
| Sagheer (40) | **SH.S.A** | Y | Y | N | N | U | N | Y | Y | Moderate |
|  |  |  |  |  |  |  |  |  |  |  |
| Moon (41) | **SH.S.A** | Y | Y | Y | Y | Y | Y | Y | Y | High |
|  |  |  |  |  |  |  |  |  |  |  |
| Masood (42) | **SH.S.A** | Y | U | N | N | N | N | Y | Y | Moderate |
|  |  |  |  |  |  |  |  |  |  |  |
| Ibrahim (43) | **SH.S.A** | Y | Y | Y | Y | Y | Y | Y | Y | High |
|  |  |  |  |  |  |  |  |  |  |  |
| Shahid (44) | **SH.S.A** | Y | Y | Y | Y | Y | Y | Y | Y | High |
|  |  |  |  |  |  |  |  |  |  |  |
| Ali (45) | **SH.S.A** | Y | Y | Y | Y | Y | Y | Y | Y | High |
|  |  |  |  |  |  |  |  |  |  |  |
| Sultan (46) | **SH.S.A** | Y | Y | Y | Y | Y | Y | Y | Y | High |
|  |  |  |  |  |  |  |  |  |  |  |
| Siddique (47) | **SH.S.A** | Y | Y | Y | Y | U | U | Y | Y | Moderate |
|  |  |  |  |  |  |  |  |  |  |  |
| Shaheen (48) | **SH.S.A** | Y | Y | Y | Y | U | U | Y | Y | Moderate |
|  |  |  |  |  |  |  |  |  |  |  |
| Malik (49) | **SH.S.A** | Y | Y | Y | Y | Y | Y | Y | Y | High |
|  |  |  |  |  |  |  |  |  |  |  |
| Jahan (50) | **SH.S.A** | Y | Y | Y | Y | U | U | Y | Y | Moderate |
|  |  |  |  |  |  |  |  |  |  |  |
| Zafar (51) | **SH.S.A** | Y | Y | N | N | U | N | Y | Y | Moderate |
|  |  |  |  |  |  |  |  |  |  |  |
| Pervez (52) | **SH.S.A** | Y | Y | Y | Y | U | U | Y | Y | Moderate |
|  |  |  |  |  |  |  |  |  |  |  |
| Afzal (53) | **SH.S.A** | Y | Y | Y | Y | U | U | Y | Y | Moderate |
|  |  |  |  |  |  |  |  |  |  |  |
| Akram (54) | **SH.S.A** | Y | Y | N | N | U | U | Y | Y | Moderate |
|  |  |  |  |  |  |  |  |  |  |  |
| Gillani (55) | **SH.S.A** | Y | Y | Y | Y | Y | Y | Y | Y | High |
|  |  |  |  |  |  |  |  |  |  |  |
| Manan(56) | **SH.S.A** | Y | Y | N | N | U | U | Y | Y | Moderate |
|  |  |  |  |  |  |  |  |  |  |  |
| Manan (56) | **SH.S.A** | Y | Y | N | N | U | U | Y | Y | Moderate |
|  |  |  |  |  |  |  |  |  |  |  |
| Khalid (57) | **SH.S.A** | Y | Y | Y | Y | Y | Y | Y | Y | High |
|  |  |  |  |  |  |  |  |  |  |  |
| Khanzada (58) | **SH.S.A** | Y | Y | Y | Y | Y | Y | Y | Y | High |
|  |  |  |  |  |  |  |  |  |  |  |
| Shaikh (59) | **SH.S.A** | Y | Y | Y | Y | U | U | Y | Y | Moderate |
|  |  |  |  |  |  |  |  |  |  |  |
| Rehman (60) | **SH.S.A** | Y | Y | Y | Y | Y | Y | Y | Y | High |
|  |  |  |  |  |  |  |  |  |  |  |
| Butt (61) | **SH.S.A** | Y | Y | N | N | U | U | Y | Y | Moderate |
|  |  |  |  |  |  |  |  |  |  |  |
| Qaiser (62) | **SH.S.A** | Y | Y | Y | Y | Y | Y | Y | Y | High |
|  |  |  |  |  |  |  |  |  |  |  |
| Ishaq (63) | **SH.S.A** | Y | Y | Y | Y | Y | Y | Y | Y | High |
|  |  |  |  |  |  |  |  |  |  |  |
| Yaseen (64) | **SH.S.A** | Y | Y | U | U | U | U | Y | Y | Low |
|  |  |  |  |  |  |  |  |  |  |  |
| Basit (65) | **SH.S.A** | Y | Y | Y | Y | Y | Y | Y | Y | High |
|  |  |  |  |  |  |  |  |  |  |  |
| Nawazish (66) | **SH.S.A** | Y | Y | Y | Y | U | U | Y | Y | Moderate |
|  |  |  |  |  |  |  |  |  |  |  |
| Memon (67) | **SH.S.A** | Y | Y | Y | Y | U | U | Y | Y | Moderate |
|  |  |  |  |  |  |  |  |  |  |  |
| Ghauri (68) | **SH.S.A** | Y | Y | Y | Y | U | U | Y | Y | Moderate |
|  |  |  |  |  |  |  |  |  |  |  |
| Saleem (69) | **SH.S.A** | Y | Y | Y | Y | U | U | Y | Y | Moderate |
|  |  |  |  |  |  |  |  |  |  |  |
| Shahid (70) | **SH.S.A** | Y | Y | Y | Y | U | U | Y | Y | Moderate |
|  |  |  |  |  |  |  |  |  |  |  |
| Saadat (71) | **SH.S.A** | Y | Y | Y | Y | U | U | Y | Y | Moderate |
|  |  |  |  |  |  |  |  |  |  |  |
| Mehmood (72) | **SH.S.A** | Y | Y | Y | Y | U | U | Y | Y | Moderate |
|  |  |  |  |  |  |  |  |  |  |  |
| Singla (73) | **SH.S.A** | Y | Y | Y | Y | U | U | Y | Y | Moderate |
|  |  |  |  |  |  |  |  |  |  |  |
| Feroz (74) | **SH.S.A** | Y | Y | Y | Y | Y | Y | Y | Y | High |
|  |  |  |  |  |  |  |  |  |  |  |
| Waris (75) | **SH.S.A** | Y | Y | Y | Y | Y | Y | Y | Y | High |
|  |  |  |  |  |  |  |  |  |  |  |

1. Were the criteria for inclusion in the sample clearly defined?

2. Were the study subjects and the setting described in detail?

3. Was the exposure measured in a valid and reliable way?

4. Were objective, standard criteria used for measurement of the condition?

5. Were confounding factors identified?

6. Were strategies to deal with confounding factors stated?

7. Were the outcomes measured in a valid and reliable way?

8. Was appropriate statistical analysis used?

11.	Awan S, Shafqat S, Kamal AK, Sonawalla A, Siddiqui S, Siddiqui F, et al. Pattern of neurological diseases in adult outpatient neurology clinics in tertiary care hospital. BMC research notes. 2017;10:1-6.

12.	Ijaz T, Syed S, Ashraf HS, Fatima M, Shehzadi H, Ahmad M. Association of Restless Leg Syndrome with Sleep Quality and Fatigue in Chronic Kidney Disease Patients with and without Hemodialysis: A Cross-sectional Study: Association of Restless Leg Syndrome with Sleep Quality and Fatigue. The Healer Journal of Physiotherapy and Rehabilitation Sciences. 2023;3(4):461-8.

17.	Sikandar R, Khealani BA, Wasay M. Predictors of restless legs syndrome in pregnancy: a hospital based cross sectional survey from Pakistan. Sleep medicine. 2009;10(6):676-8.

18.	Ishaq M, Sualeh Muhammad J, Hameed K. Risk of restless legs syndrome in low socioeconomic rheumatoid arthritis patients. Modern rheumatology. 2013;23:705-8.

19.	Shaikh RA, Solangi S, Rathi SK, Shaikh QH. Prevalence of restless legs syndrome in haemodialysis patients. JLUMHS. 2014;13(01):18.

20.	Jamal Y, Siddiqui UA, Raza A, Abbas K. Restless legs syndrome in end stage renal disease patients undergoing maintenance hemodialysis. Pakistan Armed Forces Medical Journal. 2014(4):600.

21.	Haider I, Anees M, Shahid SAH. Restless legs syndrome in end stage renal disease patients on haemodialysis. Pakistan journal of medical sciences. 2014;30(6):1209.

22.	Fasih A, Abdullah KN, Iqbal S. Obstetric restless legs syndrome in industrialized area of Pakistan. Journal of Bahria University Medical and Dental College. 2015;5(2):69-72.

23.	Siddiqi SA, Rauf J, Haroon A, Ahmed B, Kidwai SS, Nazir L. Restless legs syndrome in patients with type 2 diabetes mellitus. Pakistan Journal of Neurological Sciences (PJNS). 2015;10(1):14-20.

24.	Mahmood K, Farhan R, Surani A, Surani AA, Surani S. Restless Legs Syndrome among Pakistani Population: A Cross‐Sectional Study. International scholarly research notices. 2015;2015(1):762045.

25.	Choudhary FR, Rasheed S. PREVALENCE AND SEVERITY OF RESTLESS LEGS SYNDROME AMONG PHYSIOTHERAPY STUDENTS. High Altitude Cerebral And Pulmonary Edema in An Acclimatized Soldier With No Classic Symptoms–A Case Report. 2017;67:15.

26.	Abideen ZU, Mahmud SN, Mushtaq F, Farooq MU, Qasim YF, Hamid Z, et al. Association of hemodialysis inadequacy and duration with restless legs syndrome: A cross-sectional study. Cureus. 2018;10(5).

27.	Shahzad MF, Aslam R, Fiaz R. Prevalence of restless legs syndrome in pregnant females. Pak J Med Health Sci. 2018;12(1):177-9.

28.	Khan AM, Afridi MAR, Asghar M, Ikram M, Muhammad R. Complications During Hemodialysis In End Stage Renal Disease Patients In A Teaching Hospital. Journal of Postgraduate Medical Institute. 2019;33(3).

29.	Ishaq M, Riaz S, Iqbal N, Siddiqui S, Moin A, Sajjad S, et al. Prevalence of restless legs syndrome among medical students of Karachi: an experience from a developing country. Sleep disorders. 2020;2020(1):7302828.

30.	Saleem S, Ahmad A, Gillani SA, Hanif A, Sharif Z. Restless leg syndrome and its association with physical activity during pregnancy. Ann King Edw Med Univ. 2020;26(3):474-9.

31.	Memon SF, Shaikh SA. Prevalence of Restless Leg Syndrome in Hyderabad, Sindh, Pakistan. 2020.

32.	Qureshi MF, Arshad S, Deeba F, Dherwani A, Raza A, Akram A, et al. Prevalence Rate of Restless Leg Syndrome among Healthcare Students of Karachi. Sleep Disorders. 2020;2020:1-5.

33.	Naqvi IH, Ahmed J, Salim M, Ubaid M, Malik F, Afzal R, et al. Restless leg syndrome in patients with liver cirrhosis! its frequency, severity, and correlation. CNS & Neurological Disorders-Drug Targets-CNS & Neurological Disorders). 2021;20(5):465-72.

34.	Malik N, Gulzar S, Khan AR. Restless Legs Syndrome, Sleep, Fatigue and Mental Health in Patients with Cardiova-scular Diseases. Annals of King Edward Medical University. 2021;27(Special Issue (Jul-Sep)):440-6.

35.	Yaseen M, Jarullah FA, Yaqoob S, Shakeel HA, Maqsood H, Naveed S. Association of quality of life, anxiety, and depression with restless leg syndrome in the hemodialysis patients. Bmc research notes. 2021;14:1-6.

36.	Nawaz MS, Nawaz MS, Shah K-U, ul Mustafa Z, Ahmed A, Ahmed HS, et al. Prevalence and determinants of restless leg syndrome in type 2 diabetes mellitus (T2DM) in Pakistan. Diabetes & Metabolic Syndrome: Clinical Research & Reviews. 2021;15(2):525-8.

37.	Mubeen SM, Ahsan MD. Prevalence and associated factors of restless leg syndrome (RLS) in Pakistani women during pregnancy. Journal of Obstetrics and Gynaecology. 2022;42(6):1829-34.

38.	Sikandar K, Sharif F, Ahmad S, Ahmad A, Gilani A. Prevalence of restless leg syndrome and its impact on quality of life in medical students. Rawal Medical Journal. 2022;47(1):199-.

39.	Ishaq S, Anwar S, Mateen Akram SA, Usman HT, Anwar Z, editors. Frequency of Restless Legs Syndrome among End-Stage Renal Disease Patients on Maintenance Hemodialysis. Medical Forum Monthly; 2022.

40.	Sagheer B, Zahid A, Mubeen I, Jafar M, Amin F, Ali M. Severity and Impact of rest leg Syndrome on Sleep and its Causative Factors in Pregnant Females of Lahore. Pakistan Journal of Medical & Health Sciences. 2022;16(02):250-.

41.	Moon F. Risk factors associated with restless leg syndrome in hemodialysis patients. Rawal Medical Journal. 2022;47(4).

42.	Masood Z, Amjad F, Hashim A. Frequency of restless legs syndrome in patients with diabetes mellitus and hypertension. Rawal Medical Journal. 2022;47(3):776-.

43.	Ibrahim M, Zahoor IA, Javed AN, Rana AA, Ain N-U, Rashid M. Incidence Of Restless Leg Syndrome Among Medical School Faculty Members: Incidence of Restless Leg Syndrome Among Medical School Faculty Members. Pakistan BioMedical Journal. 2022:36-40.

44.	Shahid F, Javed R, Zia S, Akbar R, Riaz S, Tariq F. Prevalence of Restless Leg Syndrome in patients with Type-2 Diabetes Mellitus. THE JOURNAL OF MICROBIOLOGY AND MOLECULAR GENETICS. 2022;3(1):56-66.

45.	Ali M, Junejo AM, Kumar V, Ejaz A, Iram H, Ahmed S. Restless leg syndrome and associated factors in patients with end-stage renal disease on haemodialysis. The Professional Medical Journal. 2022;29(07):975-80.

46.	Sultan S, Nasir K, Qureshi R, Dhrolia M, Ahmad A. Association Of Bone Mineral Profile With Restless Leg Syndrome In Patients With End Stage Renal Disease. Journal of Ayub Medical College Abbottabad. 2022;34(3 (SUPPL 1)).

47.	Siddique S, Amin W, Aaraj S, Fatima T, Imran Y, Islam A. Restless leg syndrome (RLS) in end stage renal disease. Journal of Shifa Tameer-e-Millat University. 2022;5(1):28-31.

48.	Shaheen F. Frequency and Associated Factors of Restless Leg Syndrome in Pregnant Women During the Third Trimester. Journal of Health, Wellness and Community Research. 2023;1(2):23-9.

49.	Malik LA, Jabeen A, Javaid A, Chaudhary M, Waseem A, Idrees MQ. Relationship between restless leg syndrome and obesity; a cross-sectional study: restless leg syndrome and obesity. The Healer Journal of Physiotherapy and Rehabilitation Sciences. 2023;3(6):603-10.

50.	JAHAN N, Mehmood A, Khan N, Khan F. Effect of Restless leg syndrome on the sleep quality of medical students of university in rural Sindh. International Annals of Health Sciences. 2024;1(2).

51.	Zafar A, Qureshi AM, Tanveer S, Ghaffar E. Correlation Between Restless Legs Syndrome and Anxiety Among Pregnant Women. Yashfeen Journal of Health Innovation and Practice. 2023;1(01):1-12.

52.	Jamal MN. Original article restless legs syndrome: difference in quality of life parameters between hemodialysis patients with and without restless legs syndrome. Journal of akhtar saeed medical & dental college. 2023;5(02):84-9.

53.	Afzal MT, Farwa U, Attiq M, Jamil N, Anjum N, Bibi M. The Frequency of Restless Legs Syndrome Among University Students. Journal Riphah College of Rehabilitation Sciences. 2023;11(03).

54.	Akram A, Qasim M, Shahid N, Kazmi T, Farhat S, editors. Prevalence Of Sleep Disorders Among Medical Students Of Lahore and Their Correlation with Obesity. Proceedings; 2023.

55.	Gillani SFUHS, Iftikhar S, Bajwa AF, Baloch TA. Prevalence and severity of restless leg syndrome in patients with spinal cord injuries. JPMA The Journal of the Pakistan Medical Association. 2023;73(3):471-5.

56.	Manan A, Ahmad Z, Aijaz W, Kamran M, Memon FA, Ahmed F. Frequency of Restless Leg Syndrome in Diabetic Patients and it's Impact on Quality of Life. Pakistan Journal of Medical & Health Sciences. 2023;17(03):739-.

57.	Khalid T, Afzal MF, Amjad A. Evaluation of Restless Leg Syndrome in Patients with Type 2 Diabetes Mellitus. Iranian journal of diabetes and obesity. 2023.

58.	Khanzada SK, Bibi B, Arshad A, Iram S, Aslam I, Tabassum A, et al. Prevalence of Restless Legs Syndrome Among Madrassa Students in Jhelum, Pakistan: RLS Prevalence in Madrassa Students. Journal of Health and Rehabilitation Research. 2024;4(3).

59.	Shaikh NJ, Mahmood A, Ahsan N, Khan F. Prevalence of Restless Legs Syndrome and Associated Factors Among University IT Students in Mirpurkhas, Sindh: Prevalence of RLS Among IT Students. Journal of Health and Rehabilitation Research. 2024;4(3):1-4.

60.	Rehman A, Karim A, Ejaz M, Imran H, Iqbal Z, Aslam R, et al. Association of restless leg syndrome with fatigue and sleep quality among students taking paracetamol. Journal of Contemporary Pharmacy. 2024;8(1):22-7.

61.	Butt NI, Ghoauri MSA, Waris U, Sabeh D, Qaisar F. Frequency of Restless Legs Syndrome in Pakistani Patients With Type 2 Diabetes Mellitus. Cureus. 2024;16(6).

62.	Qaiser N, Manzoor S, Zakaullah I, Khan M, Fazal N. The Frequency and Associated Factors of Restless Leg Syndrome among Pregnant Women in 3rd Trimester. Journal of Health and Rehabilitation Research. 2024;4(2):608-12.

63.	Ishaq M, Mahesh A, Riaz SU, Samad AM, Khwaja RA, Nawab F. Restless Leg Syndrome In Pregnancy: An Experience From Low Socio-Economic Population Of Karachi. ANNALS OF ABBASI SHAHEED HOSPITAL AND KARACHI MEDICAL & DENTAL COLLEGE. 2024;29(4):420-6.

64.	Yaseen A, Yasin S, Haq ZU, Ahmad I, Hussain M, Pervez N, et al. Frequency of Sleep Disorders in Rheumatoid Arthritis Patients. Cureus. 2024;16(10).

65.	Basit B, Sabir O, Shahid AH, Kanwal A, Qadri GA, Rizvi N. Evaluation of Restless Leg Syndrome in Patients with Non-Dialysis Dependent Chronic Kidney Disease at Fatima Memorial Hospital Lahore: A Cross-sectional Study. Life and Science. 2024;5(1):06-.

66.	Nawazish M, Saeed I, Jamil MI, Nadeem M. Frequency of Restless Legs Syndrome in Hemodialysis Patients. Indus Journal of Bioscience Research. 2025;3(7):755-9.

67.	Memon Z, Nisar N. Frequency and risk factors of restless leg syndrome in pregnant women. Journal of Shalamar Medical & Dental College-JSHMDC. 2025;6(1):48-52.

68.	Ghauri MI, Iqbal AM, Riaz SU, Rasool T, Khan N, Ishaq K, et al. Restless leg syndrome in osteoarthritis: A common but overlooked combination-A study in south Asian population. Journal of Orthopaedic Reports. 2025;4(2):100637.

69.	Saleem A, Mahmood A, Qayyum M, Islam F, Waseem M, Younus R. Association of Various Factors with Restless Leg Syndrome in Patients of Chronic Kidney Disease. Pakistan Armed Forces Medical Journal. 2025;75(4):674.

70.	Shahid A, Zafar F, Javed A, Qayyum F, Fatima B. Association of restless leg syndrome with physical activity and musculoskeletal pain among medical students. Greenfort International Journal of Applied Medical Science. 2025;3(4):213-6.

71.	Saadat Y, Khan MI, Ali S, Jamali AH, Mushtaq F, Jabeen A. Evaluation of Sleep Disorders in Patients with Chronic Kidney Disease. Journal of Bahria University Medical and Dental College. 2025;15(04):390-6.

72.	Mehmood N, Iqbal M, Raheem R, Chandran SP, Ahmed W. Prevalence of Restless Legs Syndrome in Patients with Type 2 Diabetes and Its Impact on Daytime Sleepiness: Restless Legs Syndrome in Patients with Type 2 Diabetes. THE THERAPIST (Journal of Therapies & Rehabilitation Sciences). 2025:27-31.

73.	Singla B, Abbas SF, Khan MN, Amir M, Cheema MU, Akhlaq MA, et al. Exploring the Link Between Caffeine Intake, Sleep Quality, and Restless Legs Syndrome Among Medical Students. Cureus. 2025;17(5).

74.	Feroz S, Khan L, Anwar SB, Fatima A, Samson M, Parkash K. Gestational Neuro-Otological Manifestation Patterns Across Trimesters in Karachi, Pakistan: A Retrospective Cross-Sectional Study. Cureus. 2025;17(12).

75.	Waris B, Butt NI, Khizar I, Waris U, Baber A, Javed U. Association of Restless Legs Syndrome with Hypertension and its Complications: A Cross-Sectional Study: RLS, Hypertension, and Related Complications. Pakistan Journal of Rehabilitation. 2026;15(1):26-30.
