# Supplementary material for: Prevalence of restless legs syndrome in Pakistan: a systematic review and meta-analysis across diverse study populations
Source: BMC Neurol. 2026 Jun 15;26:537. doi: 10.1186/s12883-026-04981-8 (PMC13501774; doi:10.1186/s12883-026-04981-8)
Supplement: Supplementary file 3 [file 12883_2026_4981_MOESM3_ESM.docx]

**Search Strategy**

Search Date: 1 Feb 2026

**1. Ovid MEDLINE(R) ALL 1946 to March 12, 2025**

| 1 | exp Restless Legs Syndrome/ or ("restless Leg*" or "ekbom disease" or "ekbom syndrome" or RLS).ti,ab. | 8755 |
| --- | --- | --- |
| 2 | exp Pakistan/ or Pakistan.in. or Pakistan*.ti,ab. | 118142 |
| 3 | 1 and 2 | **45** |

(exp Restless Legs Syndrome/ or ("restless Leg*" or "ekbom disease" or "ekbom syndrome" or RLS).ti,ab.) and (exp Pakistan/ or Pakistan.in. or Pakistan*.ti,ab.)

**2. Embase**

| #1 | 'restless legs syndrome'/exp OR ('restless Leg*' OR 'ekbom disease' OR 'ekbom syndrome' OR RLS):ti,ab | 17,055 |
| --- | --- | --- |
| #2 | Pakistan/exp OR Pakistani/exp OR Pakistan:ff OR Pakistan*:ti,ab | 66,871 |
| #3 | #1 AND #2 | **25** |

**3. Scopus**

| #1 | TITLE-ABS-KEY("restless Leg*" OR "ekbom disease" OR "ekbom syndrome" OR RLS) | 24,793 |
| --- | --- | --- |
| #2 | TITLE-ABS-KEY(Pakistan*) | 126,782 |
| #3 | #1 AND #2 | **23** |

**4. Web of Science Core Collection (SCIE, SSCI, and ESCI)**

| #1 | TS=("restless Leg*" OR "ekbom disease" OR "ekbom syndrome" OR RLS) | 16,216 |
| --- | --- | --- |
| #2 | TS=Pakistan* | 90,846 |
| #3 | #1 AND #2 | **22** |

| 1 | Medline | 45 |
| --- | --- | --- |
| 2 | Embase | 25 |
| 3 | Scopus | 23 |
| 4 | Web of Science Core Collection | 22 |
| Total | | **115** |
